# Isolation and possibility of vertical transmission of G9P[23] and G12P[7] group A rotavirus strains in pigs

**DOI:** 10.1186/s40813-025-00445-6

**Published:** 2025-06-06

**Authors:** Yang Li, Chunliu Gao, Lili Wu, Jie Qing, Minxia Zhang, Mengli Qiao, Zhiqiang Hu, Bingzhou Zhang, Chen Yang, Zewei Wang, Lulu Li, Zheng Yan, Weisheng Wu, Wei Liu, Jing Ren, Xiaowen Li

**Affiliations:** 1 Shandong Engineering Research Center of Pig and Poultry Health Breeding and Important Disease Purification, Shandong New Hope Liuhe Co., Ltd, Qingdao, China; 2 Juye Xinhao Agriculture and Animal Husbandry Co., Ltd, Heze, China; 3 Xiajin New Hope Liuhe Agriculture and Animal Husbandry Co., Ltd, Dezhou, China; 4 Swine Health Data and Intelligent Monitoring Project Laboratory, Dezhou University, Dezhou, China; 5 Key Laboratory of Animal Epidemic Disease Detection and Prevention in Panxi District, College of Animal Science, Xichang University, Xichang, China

**Keywords:** Pig, Rotavirus, Vertical transmission, G9P[23], G12P[7]

## Abstract

**Background:**

Porcine group A rotavirus (RVA) is a significant causative agent of diarrhea in piglets, leading to substantial economic losses in pig farms worldwide. While horizontal transmission of RVA among pig populations is well documented, the possibility of vertical transmission from sows to newborn piglets has not been definitively confirmed.

**Results:**

In this study, piglet testicles, umbilical cord blood, and colostrum were collected from porcine RVA (PoRVA)-active farms for analysis. The samples presented high PoRVA-positive rates, with 70.00% in the testicle samples, 55.00% in the umbilical cord blood samples, and 73.33% in the colostrum samples. Immunohistochemical assays confirmed the presence of PoRVA in neonatal piglet testicles. Additionally, two PoRVA strains, RVA/Pig/CHN/QT/2023/G9P [23] (QT2023) and RVA/Pig/CHN/BH/2023/G12P [7] (BH2023), were isolated from newborn piglet testicles. Complete genome analyses revealed that strains QT2023 and BH2023 both presented a Wa-like backbone, with the genotype constellation of G9-P [23]-I5-R1-C1-M1-A8-N1-T1-E1-H1 and G12-P [7]-I5-R1-C1-M1-A8-N1-T1-E1-H1, respectively. While strains QT2023 and BH2023 originated from PoRVAs, sequence identities and phylogenetic analyses suggested close relationships with human rotaviruses in specific genes. Furthermore, successful viral replication of these strains in MA-104 cells was observed. Inoculation of PoRVA-negative piglets with strains QT2023 and BH2023 resulted in clinical diarrhea, fecal virus shedding, and intestinal pathological changes, highlighting the pathogenicity of these strains.

**Conclusion:**

This study provides evidence that PoRVA can breach the placental barrier and spread to newborn piglets through vertical transmission. These discoveries enhance our understanding of the transmission route of porcine RVA and have the potential to guide the development of efficient vaccine strategies for combating this disease.

## Background

Rotavirus (RV), a member of the *Reoviridae* family, is now recognized as a major cause of acute gastroenteritis in young animals and children. While RV infection in adults is typically asymptomatic due to postinfection immunity, immune system maturation, and physiological changes in the gut, it can cause vomiting, diarrhea, and dehydration in young animals and children [1–3]. Severe diarrhea due to RV infection results in 440,000 deaths among children less than 5 years of age, and most deaths occur in developing countries [4]. The first discovery of RV occurred in 1969 in calves, where it was identified as the primary causative agent of calf diarrhea [5]. RV infection subsequently occurred in humans, pigs, and other animals in the 1970s [6, 7]. The occurrence of independent porcine-to-human zoonotic transmission events has been reported [8]. RV has become a global concern for public health and veterinary health and has spread widely in many countries [9].

RV is a nonenveloped double-stranded RNA (dsRNA) virus with a genome consisting of eleven segments encoding six structural viral proteins (VP1-VP4, VP6, and VP7) and six nonstructural proteins (NSP1-NSP6). RVs are classified into ten groups (A-J) on the basis of the antigenic relationships of the VP6 sequences [10]. Groups A, B, and C are the most common groups that infect humans and other animals [9]. Rotavirus A species (RVA), RVB, RVC, and RVH are four species of RVs that are prevalent in pig herds [11]. Group A of PoRV (PoRVA) is responsible for causing piglet diarrhea before and after weaning. It accounts for more than 90% of the diarrhoeal diseases induced by PoRV on commercial pig farms [12]. RVAs are classified into different genotypes with the notations Gx-P[x]-Ix-Rx-Cx-Mx-Ax-Nx-Tx-Ex-Hx used for the VP7-VP4-VP6-VP1-VP2-VP3-NSP1-NSP2-NSP3-NSP4–NSP5 encoding genes, respectively [9]. The genes of human RVs normally have Wa-like (I1-R1-C1-M1-A1-N1-T1-E1-H1), DS-1-like (I2-R2-C2-M2-A2-N2-T2-E2-H2), or AU-1-like (I3-R3-C3-M3-A3-N3-T3-E3-H3) genotype constellations [13].

The genetic diversity, epidemiology and pathogenicity of PoRVAs have been well studied [9]. Rotavirus G-types and P-types, depending on the sequence diversities of VP7 and VP4, respectively, elicit neutralizing antibody responses [14]. To date, at least 36 G-types and 51 P-types have been detected in humans and animals. The high degree of genetic and antigenic variation among rotaviruses poses challenges to the development of effective vaccine programs in both human and animal populations [9, 15]. Moreover, PoRV efficiently spreads through fecal‒oral routes, with pigs being susceptible to infection through direct or indirect exposure to virus particles [16]. Following infection, the RV predominantly replicates in the villous epithelial cells of the small intestine, leading to shedding of the intestinal villi. The potential causes of diarrhea triggered by RV in pigs and humans include (i) malabsorption resulting from damage to intestinal cells; (ii) the release of vasoactive substances by infected epithelial cells, leading to villi ischemia and the activation of the enteric nervous system; and (iii) the intracellular or extracellular activity of the RV nonstructural protein NSP4, which stimulates intestinal secretion [16].

PoRVA commonly infects pigs between 1 and 5 weeks of age, with piglets aged 7 to 21 days experiencing the most severe diarrhea symptoms. However, in some practical pig farming scenarios, diarrhea symptoms caused by PoRVA infection have been occasionally observed in suckling piglets aged 1–3 days. Rotaviruses can escape the gastrointestinal tract in children and young animals, resulting in antigenaemia and possibly viraemia [17–19]. Accordingly, we suspected that PoRVA can be transmitted from sows to piglets during pregnancy. The findings of vertical transmission of PoRVA in this study provide valuable insights into prevention and control strategies for PoRV in pig production.

## Materials and methods

### Sampling

The study was conducted on two medium-sized local farms (farm QT in Zhejiang Province and farm BH in Shandong Province, China) known for their endemic PoRV infections, each housing approximately 500 sows. These farms utilize a farrow-to-finish production system and maintain strict biosecurity measures, following an all-in/all-out approach. During sampling, approximately 50–70% of the piglets exhibited signs of diarrhea, with symptoms typically manifesting between 5 and 10 days of age. Notably, around 20% of the piglets displayed diarrhea within just 2 days of birth. Sixty sets of samples of piglet testicles, umbilical cord blood, and colostrum were collected immediately after birth. To collect the testicles, the skin was cleaned with 5% sodium hypochloride (NaClO) to degrade the potential PoRV nucleic acids. The skin was then scratched via a sterile blade, and the testicles were carefully removed to resealable the bags to prevent cross-contamination. To collect umbilical cord blood, the outer surface of the undetached umbilical cord was disinfected with 5% NaClO. The umbilical cord was subsequently cut with disinfected scissors. The blood was collected in a sterile centrifuge tube and then centrifuged at 3,000 × *g* for 2 min to separate the serum. To collect colostrum, the nipple was disinfected with 5% NaClO. The colostrum was squeezed directly into a sterile centrifuge tube. All the collected samples were frozen at -80 °C until further detection.

### Quantification of viral RNA loading by qRT‒PCR

The frozen testicles were transferred to liquid nitrogen and crushed to powder with a pestle. The viral loads in the testicles, umbilical cord blood, and colostrum samples were determined via reverse transcription qPCR (qRT‒PCR). First, viral RNA extraction was performed via the FastPure Complex Tissue/Cell Total RNA Isolation Kit (Vazyme, Nanjing, China) following the instructions of the manufacturers. The TransScript Probe One-Step qRT‒PCR Kit (TransGen, Beijing, China) was then used for the qPCR. The specific primers and probes used for detecting viral nucleic acids are listed in Table 1. The qPCR protocol involved an initial reverse transcription step at 45 °C for 5 min followed by a predenaturation step at 94 °C for 30 s. The qPCR cycling conditions consisted of 40 cycles, with denaturation at 94 °C for 5 s and annealing extension at 60 °C for 30 s. Samples with a CT value of less than 35 were classified as positive for RV. If a sample’s CT value in the initial test was greater than 35 but less than 40, a second test was performed. A CT value of less than 40, along with a typical amplification curve in the second test, was also considered positive for RV.

### Standard curve

To construct the standard curve, partial sequences of the NSP3 gene were amplified by PCR and the products were cloned into pMD18-T plasmids (D101A, Takara, Japan) as previously described [20]. The standard curve was constructed using logarithmic 10-fold dilutions ranging from 5 × 10^7^ to 5 genome copies. The PoRV genome copy numbers corresponding to the copy numbers of the standard plasmid were calculated using the following formula:

$$\:Copy\:number=\frac{\text{m}\times\:6.022\times\:{10}^{23}}{\text{1,842,393.52}\times\:1\times\:{10}^{9}}$$

where m (/g) is the quality of plasmids measured using a BioSpecnano Micro-volume UV-Vis Spectrophotometer (Shimadzu, Kyoto, Japan), 6.022 × 10^23^ is the Avogadro number, 1,842,393.52 (Da) is the molecular weight of standard plasmids calculated using the Sequence Manipulation Suite [21], and 1 × 10^9^ is used to convert the molecular weight of the plasmids to nanograms. The CT values obtained from qPCR analysis were then converted to PoRV genome equivalents according to the standard curve.

**Table 1:** Sequence of primers used in the study

Target gene	Primer/probe		Primer sequence (5’-3’)
PEDV M		F R P	CATCTGATTCTGGACAGTTG CTATACACCAACACAGGCTC FAM-TTTCAGAGCAGGCTGCATAT-BHQ1
TGEV N		F R P	TGTTTGGAAGCTATTGGACTT TTAGGATCATCCTTTGGCAAG FAM-AAGATGGCGACCAGATAGAAGTCACG-BHQ1
PDCoV N		F R P	AACAGCAGAAGAAACCTAAAA GCTGATTCCTGCTTTATCTCA FAM-TGCCAGCAGACAAACAGGA-BHQ1
PoRVA NSP3		F1 R1 P	ACCATCTACACATGACCCTCTATGAG ACATAACGCCCCTATAGCCATTTAG FAM-ACAATAGTTAAAAGCTAACACTG-BHQ1
PoRVA VP1		F	GGCTATTAAAGCTGTACAATGG
		R	GGTCACATCTAAGCRCTC
PoRVA VP2		F	GGCTATTAAAGGYTCAATGG
		R	GGTCATATCTCCACAGTGG
PoRVA VP3		F1	TACCTCTGATGGTGTAAGCATGA
		R1	GGAGTGTCTAATGGGTCCCACGT
		F2	TATACATGGTTGGCTCAGC
		R2	CCTAAACATCCATCATATGA
		F3	GAGAACGTATTTATACAACCTCC
		R3	GGAATAAACATACGTGATTGTCC
		F4	CAGAACTATTTAAAATGCAAAT
		R4	TTAGCTCACTCAGACATATCAA
PoRVA VP4		F	GGCTATAAAATGGCTTCGCTCA
		R	TRCTTAYARTCTACATTGCA
PoRVA VP6		F	GGCTTTWAAACGAAGTCTTC
		R	GGTCACATCCTCTCACTA
PoRVA VP7		F	GGCTTTAAAAGAGAGAATTTC
		R	GGTCACATCATACAGTTCTAAC
PoRVA NSP1		F	ATGAAAAGTCTTGTRGAAGCC
		R	CCTAGGCGCTACTCTAGTGC
PoRVA NSP2		F	GAGCCTTGCGGTGTAGCCATG
		R	GGTCACATAAGCGCTTTCT
PoRVA NSP3		F2	GATGGAGTCTACTCAGCAGATGG
		R2	CTATTGTGCTCATAGAGGGTC
PoRVA NSP4		F	AGTTCTGTTCCGAGAGAGCG
		R	TTCCTTCCATTAACGTCCAAC
PoRVA NSP5		F	GGCTTTTAAAGCGCTACAGTG
		R	ATCTTCGATCAATTGCATTGC

Note: R, A/G; W, A/T; Y, C/T. F, forward primer; R, reverse primer; P, probe. PoRVA NSP3-F1/R1/P was used for qRT‒PCR. PoRVA NSP3-F2/R2 was used for RT‒PCR. PoRVA VP3 was divided into four parts for amplification via four pairs of primers.

## Results

### Detection of PoRVA in reproduction-related samples

**Fig. 1:**
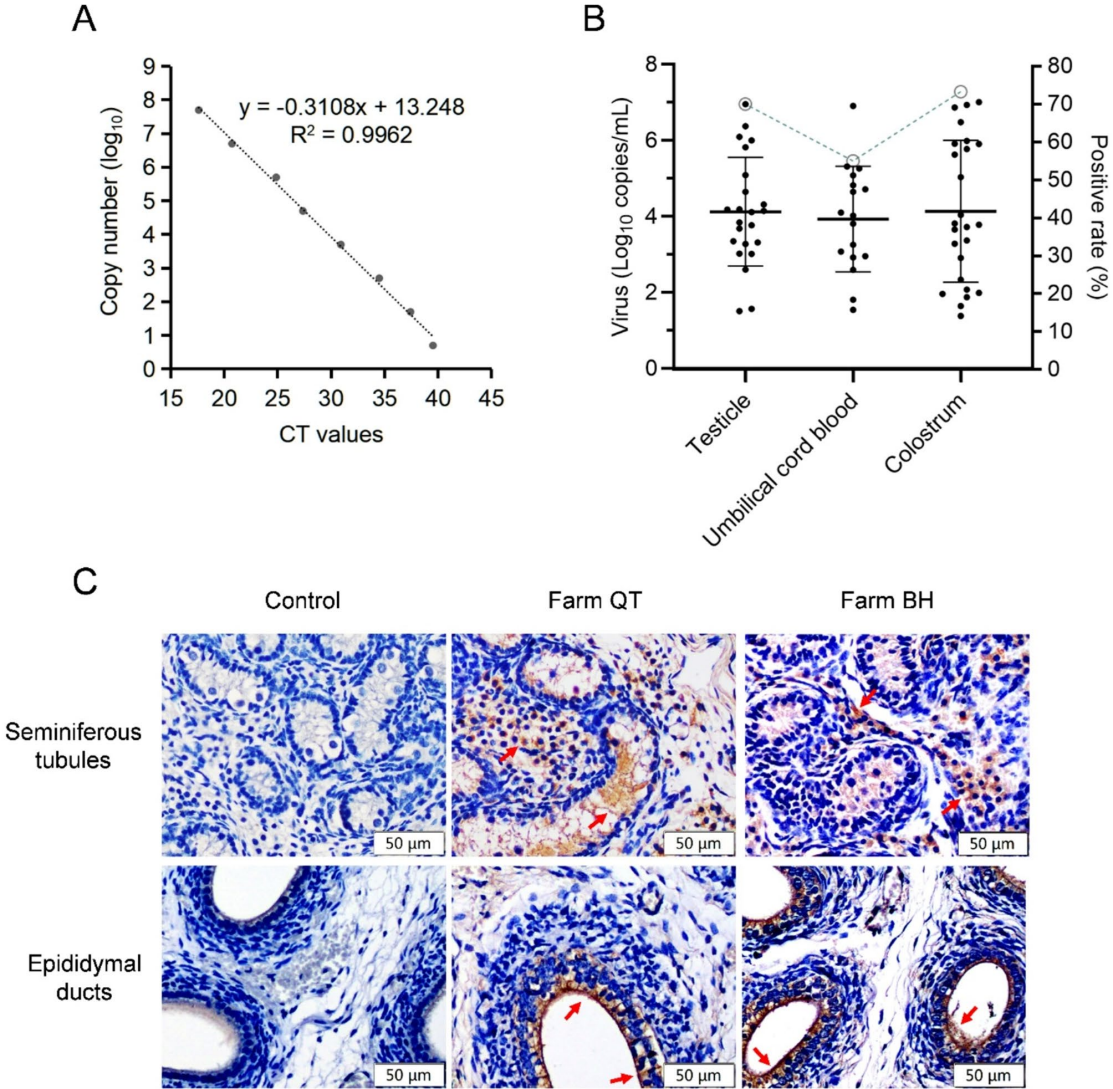
Detection of PoRV in reproduction-related samples. (**A**) Establishment of a standard curve for the qPCR assay targeting PoRV NSP3. The CT values were plotted against the logarithm of the standard plasmid copies to perform linear regression analysis. The experimental points aligned in a straight line with a high correlation coefficient (R^2^ = 0.9962), indicating accurate prediction. (**B**) Quantification of virus copies and percentage of PoRVA-positive cases among reproductive-related samples. (**C**) Immunohistochemistry (IHC) analysis of testicular tissues. Testicular tissues from newborn piglets were collected for examination. IHC was conducted using an anti-PoRV polyclonal antibody. Positive signals, indicated by brown staining and marked by arrows, confirmed the presence of the PoRV antigen within the testicular tissues. Piglets treated with saline served as negative controls

To investigate the potential transplacental transmission of PoRVA, sixty sets of piglet testicles, umbilical cord blood, and colostrum samples were collected on the day of birth from PoRVA-active farm QT in Zhejiang Province and farm BH in Shandong Province. Total RNA was extracted from the samples and subjected to qRT‒PCR using specific primers and probes (Table 1). All the samples were negative for porcine epidemic diarrhea virus (PEDV), transmissible gastroenteritis virus (TGEV), and porcine deltacoronavirus (PDCoV). To assess the sensitivity of the qPCR assay for PoRVA detection, a standard curve was generated using a 10-fold serial dilution of standard plasmids containing PoRVA NSP3 partial sequences. The detection limit of qPCR assay was determined to be approximately 6.55 copies/µL according to the linear relationship (Fig. 1A). The analysis revealed that the average PoRVA positive rate was 70.00% (42/60) in the testicle samples, 55.00% (33/60) in the umbilical cord blood samples, and 73.33% (44/60) in the colostrum samples (Fig. 1B).

### Immunohistochemical assay of PoRVA infection in newborn piglets

To further validate the presence of PoRVA in piglet testicles, three PoRVA-RNA-positive piglets were randomly selected from farms QT and BH. The immunohistochemical (IHC) assay was conducted using an anti-PoRV polyclonal antibody. In alignment with the qRT‒PCR findings, distinct brown positive signals were observed within the connective tissue between seminiferous tubules and the pseudostratified columnar epithelial cells of the epididymal ducts (Fig. 1C, representative results). These results demonstrated that PoRVA successfully colonized the testicular tissue of neonatal piglets and expressed viral proteins.

### Genotype constellation analysis

**Fig. 2:**
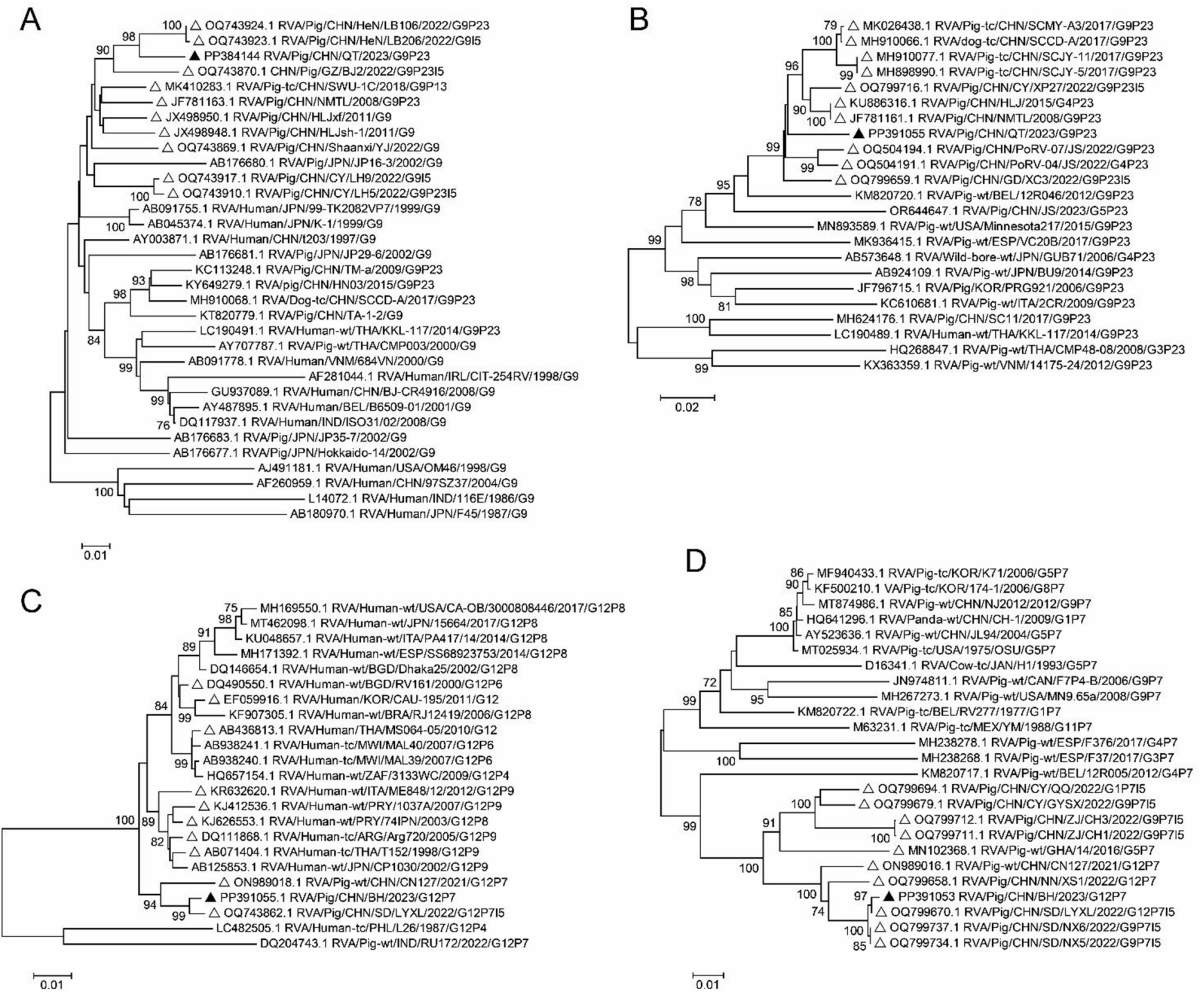
Phylogenetic tree of the VP7 and VP4 genes. Phylogenetic analyses were conducted using the sequenced strains from this study and representative strains. The numbers at the nodes indicate the level of bootstrapping based on neighbor‒joining analysis with 1,000 replications; only values above 70% are shown. The scale bar represents the number of nucleotide substitutions per site. Strains QT2023 and BH2023 are denoted by black triangles. The top 10 sequences that align significantly with strains QT2023 and BH2023 are marked with white triangles. **(A)** Phylogenetic tree of the VP7 gene of strain QT2023. **(B)** Phylogenetic tree of the VP4 gene of strain QT2023. **(C)** Phylogenetic tree of the VP7 gene of strain BH2023. **(D)** Phylogenetic tree of the VP4 gene of strain BH2023

To determine the genotype of the emerging PoRVA strains in piglet testicles, the open reading frame (ORF) sequences for all 11 genome segments were amplified and sequenced via the primers listed in Table 1. BLASTn analysis of the individual segments of the prevalent PoRV strains in farm QT revealed that the VP1 and NSP3 genes presented the highest similarity to human RVA strains. The VP3 gene displayed greater similarity to a giant panda strain. The other gene segments (VP2, VP4, VP6, VP7, NSP1, NSP2, NSP4, and NSP5) presented the highest similarity to the PoRVA strains. The strains from the farm QT breed presented 92.5–98.5% nucleotide sequence homology and 96.9–99.7% amino acid sequence homology with the most closely related strains. Accordingly, the genotype constellation was determined to be G9-P [23]-I5-R1-C1-M1-A8-N1-T1-E1-H1, and the strain was named RVA/Pig/CHN/QT/2023/G9P [23] (QT2023 for short). Additionally, the VP7 gene of strain QT2023 presented the highest nucleotide sequence identity (96.8%) with that of the Chinese porcine strain LB106 (G9P [23]) (Table 2). Phylogenetically, the VP7 gene clustered with several porcine strains from China (Fig. 2A). The VP4 gene of strain QT2023 presented the highest nucleotide sequence identity (96.8%) with those of the Chinese porcine strains HLJ2015 and NMTL2008, which clustered into one lineage (Fig. 2B).

**Table 2:** Nucleotide and amino acid identities of PoRV strains compared with the closest strains from the GenBank database

	RVA/Pig/CHN/QT/2023/G9P[23]					RVA/Pig/CHN/BH/2023/G12P[7]				
Gene	Closest strain	Nucleotide (%)	Amino acid (%)	Genotype	Accession No.	Closest strain	Nucleotide (%)	Amino acid (%)	Genotype	Accession No.
VP1	RVA/Human-wt/CHN/R1954/2013/G4P[6]	96.60	98.60	R1	KF726069.1	RVA/Human-tc/ CHN/LL3354/2013	94.90	98.70	R1	KC139781.1
VP2	RVA/Pig-wt/CHN/YN/2015	96.00	99.30	C1	KJ466983.1	RVA//pig-wt/CHN/JSJR/2023	96.80	99.10	C1	PP100150.1
VP3	RVA/Giant panda/CHN/CH-1/2008	96.30	98.40	M1	HQ641295.1	RVA/Pig-wt/KOR/PRG9121/2012	92.10	97.20	M1	JF796736.1
VP4	RVA/pig-wt/CHN/HLJ/15/1/2016	96.50	98.30	P[23]	KU886316.1	RVA/Pig-wt/CHN/SD/NX6/2022/G9P[7]I5	99.50	99.20	P[7]	OQ799737.1
VP6	RVA/Pig-wt/CHN/KY/2022	97.90	99.70	I5	OR127198.1	RVA/Pig-wt/CHN/SD/LYXL/2022/G12P[7]I5	99.80	100.00	I5	OQ799767.1
VP7	RVA/Pig/CHN/HeN/LB106/2022/G9P23I5	96.80	97.50	G9	OQ743924.1	RVA/Human-tc/ARG/Arg720/2005/G12P[9]	97.60	98.20	G12	DQ111868.1
NSP1	RVA/Pig/CHN/SC11/2017/G9P[23]	95.60	96.90	A8	MH624168.1	RVA/Pig-wt/CHN/CN127/2021/G12P[7]	96.40	98.60	A8	ON989008.1
NSP2	RVA/Pig/CHN/SD-1/2021	98.50	99.00	N1	ON676176.1	RVA//Pig-wt/CHN/JSJR/2023	97.30	99.40	N1	PP100157.1
NSP3	RVA/Human-wt/CHN/E931/2008/G4P[6]	92.50	98.40	T1	KF726041.1	RVA//Pig-wt/CHN/YT/2022	96.40	98.70	T1	OR232957.1
NSP4	RVA/Pig-tc/CHN/TM-a-P60/2018/G9	95.60	98.30	E1	MH697655.1	RVA/Pig-wt/CHN/LLP48/2008	97.70	98.30	E1	KJ126820.1
NSP5	RVA/Pig-wt/ESP/F222/2017/G9P[23]	98.10	98.40	H1	MH238174.1	RVA/Pig-wt/BGD/H14020027/G4P[49]	98.20	98.00	H1	MK227397.1

Similarly, the PoRVA strain in farm BH has a genotype of G12-P [7]-I5-R1-C1-M1-A8-N1-T1-E1-H1 and was named RVA/Pig/CHN/BH/2023/G12P [7] (BH2023 for short). The BH2023 strain presents 92.1–99.8% nucleotide sequence homology and 97.2–100% amino acid sequence homology with the most closely related strains. The VP1 and VP7 genes of strain BH2023 are closely related to genes of human-origin rotaviruses (Table 2). The VP7 gene of BH2023 has the highest nucleotide sequence identity with the Arg720 strain in Argentina (Table 2) but forms a cluster with the CN127 strain reported from the first case of G12P [7] infection in pigs in China (Fig. 2C) [15]. The VP4 gene of strain BH2023 has the highest homology (99.5%) with the porcine strain NX6 detected in China in 2022 (Table 2), and these strains form a separate lineage (Fig. 2D).

### Isolation of the emerging PoRVA strains

**Fig. 3:**
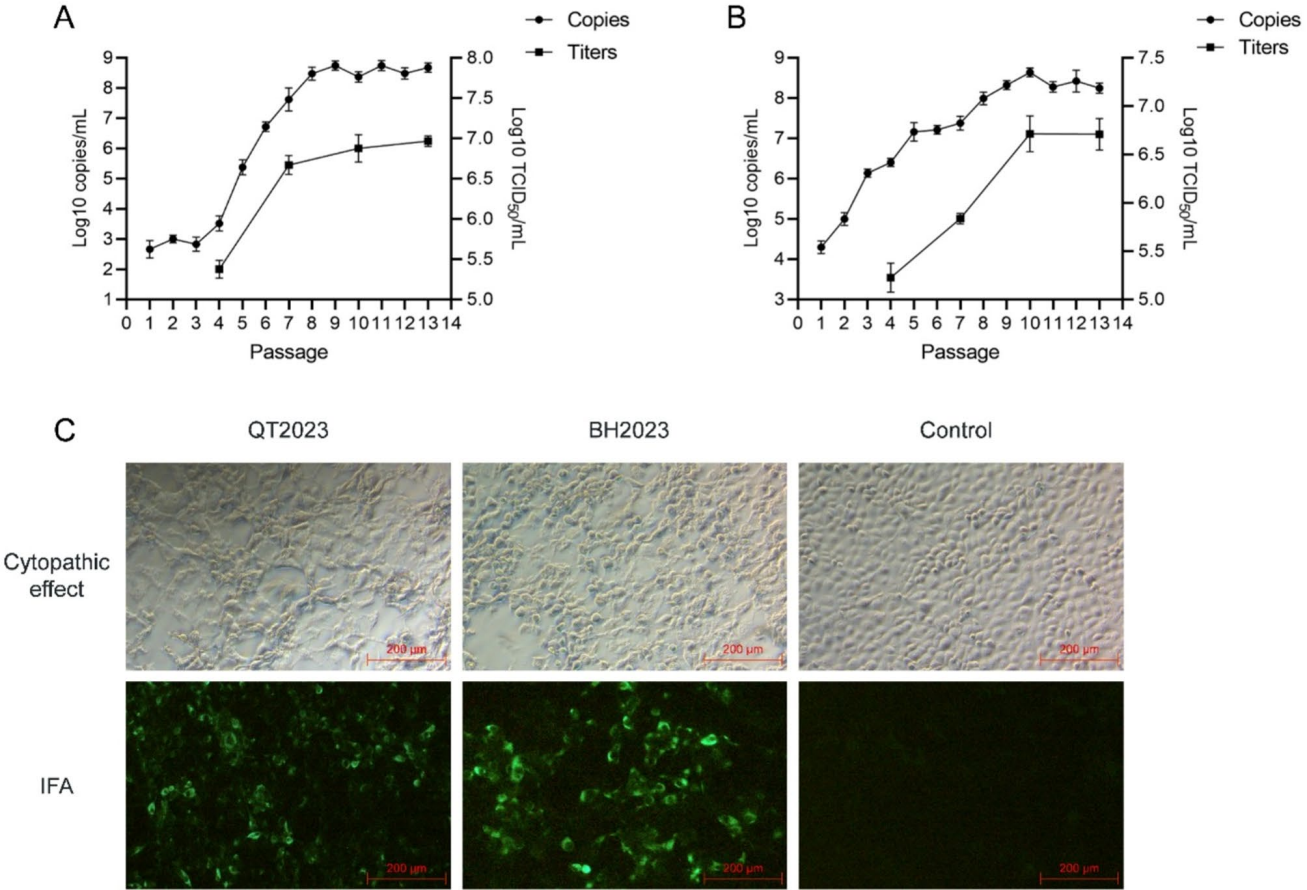
Isolation and culture of the emerging porcine RVA strains. **(A)** MA-104 cells were incubated with passages P1 to P13 of strain QT2023. The CT values of the PoRV nucleic acid were detected via qRT‒PCR. The sample CT values were subsequently converted to copy numbers on the basis of the standard curve (data not shown). The titre of PoRV (P4, P7, P10, and P13) was determined via a TCID_50_ assay. **(B)** MA-104 cells were incubated with passages P1 to P13 of strain BH2023. Similar procedures were likely followed, as shown in Fig. 3A, to detect PoRV nucleic acid and determine the PoRV titre. **(C)** MA-104 cells were incubated with passage P5 of strain QT2023 or passage P3 of strain BH2023. After 24 h, the cytopathic effects were recorded. The protein expression of PoRV was detected via an immunofluorescence assay (IFA) with an anti-PoRV polyclonal antibody

To further study the characteristics of the PoRVA strains, the viruses were isolated. First, the supernatant of newborn piglet testicles was centrifuged, filtered, and inoculated onto monolayers of MA-104 cells. Forty-eight hours later, the cells were subjected to two freeze‒thaw cycles to obtain the first passage (P1) of virus. The two strains were steadily propagated after P10 according to the steady copies. The titres of the P13 strain QT2023 and P13 strain BH2023 were determined to be 1 × 10^7.0^ TCID_50_/mL (Fig. 3A) and 1 × 10^6.75^ TCID_50_/mL (Fig. 3B), respectively. Moreover, the cells developed a visible cytopathic effect (CPE) after infection with the P5 strain QT2023 or the P3 strain BH2023. An immunofluorescence (IFA) assay using an anti-PoRV polyclonal antibody indicated that the PoRVA strains were successfully propagated in MA-104 cells (Fig. 3C). The isolated virus strains (P13) have same genotype constellations and limited mutations with their wild type strains (data not shown). These results suggested that PoRVA strains were successfully isolated from the newborn piglet testicles.

### Pathogenicity of strains QT2023 and BH2023 in piglets

**Fig. 4:**
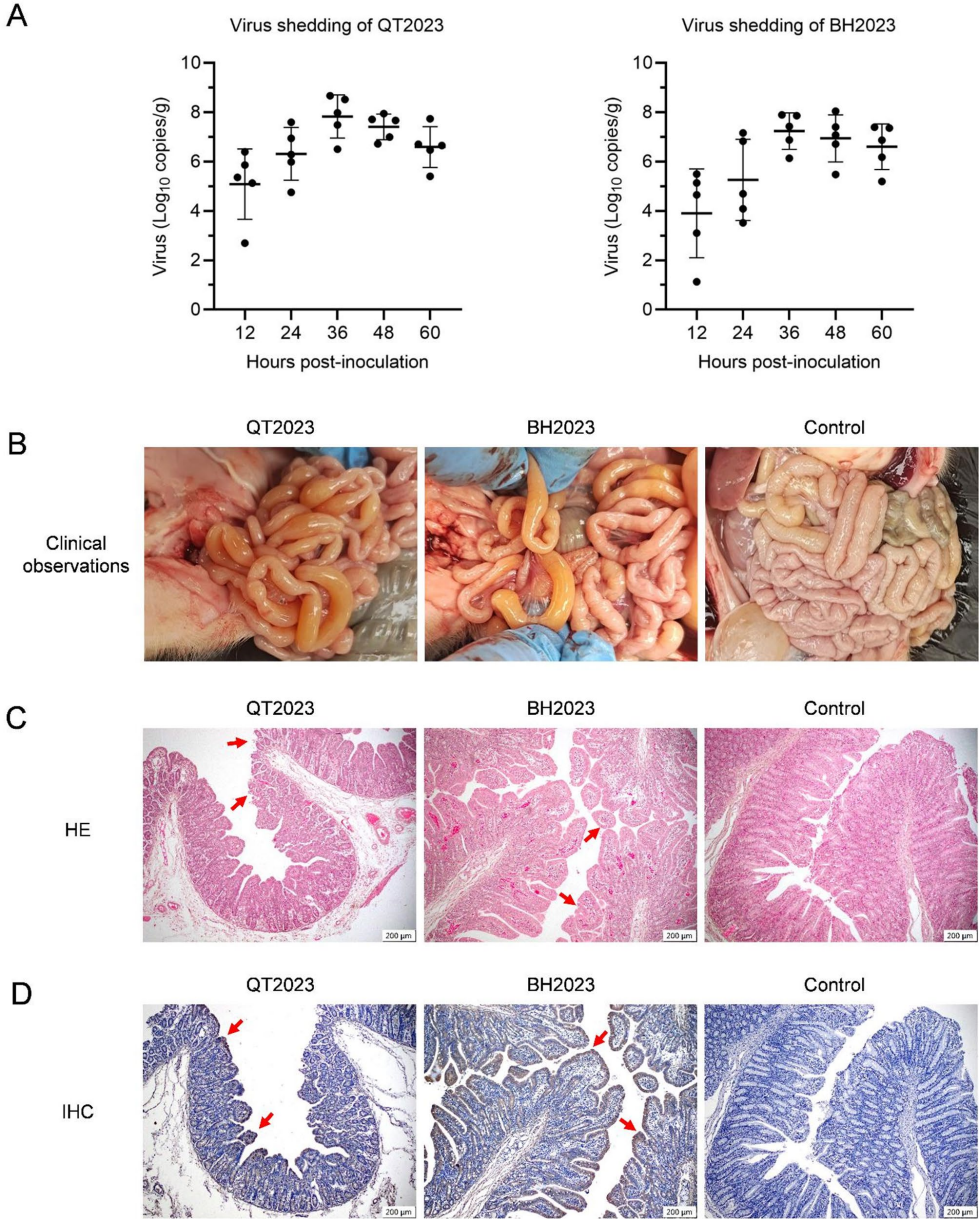
Pathogenicity of strains QT2023 and BH2023. Fifteen 7-day-old PoRV-negative suckling piglets were randomly allocated into three groups, each comprising 5 piglets. Two groups of piglets were orally challenged with strains QT2023 or BH2023. Piglets treated with saline served as the negative control in this analysis. Each group consisted of three piglets. **(A)** Fecal swabs were collected to quantify virus shedding in the feces via qRT‒PCR. **(B)** Anatomical examinations were conducted 60 hpi. Representative results are presented. **(C)** Histopathology of tissues from piglets exposed to the RVA strains mentioned was examined. Jejunum samples were collected when piglets displayed clinical symptoms. Sections of the jejunum were stained with hematoxylin and eosin for analysis. Abnormalities such as shortening and coarsening of villi, irregular striated borders, and desquamation of epithelial cells were observed and are indicated by arrows. **(D)** IHC staining of jejunum tissues was performed using a PoRV-specific polyclonal antibody. Brown positive signals, indicated by arrows, indicate the presence of PoRV in the tissues

To evaluate the pathogenicity of the isolated PoRVA strains, fifteen 7-day-old PoRV-negative piglets were orally inoculated with strains QT2023 (P13), BH2023 (P13) or saline. Fecal swabs were collected to quantify virus shedding in feces via qRT‒PCR. As a result, all inoculated piglets exhibited clinical diarrhea within 12–24 h, which persisted for 60 h, whereas mock-inoculated piglets presented no clinical signs (data not shown). Fecal virus shedding from PoRVA-inoculated piglets was detectable from 12 to 60 hpi, peaking at 36 hpi (Fig. 4A). No virus shedding was observed in mock-inoculated piglets. The piglets were subsequently euthanized and necropsied. Anatomical examinations revealed fluid accumulation, thinning of the intestinal wall, and transparency in the small intestine of infected piglets, in contrast with the normal appearance of the intestinal tract in the control group (Fig. 4B).

### Histopathological lesions of the intestine post challenge

To further explore the pathological changes in piglets infected with strains QT2023 and BH2023, jejunal samples were collected for histopathological analysis. HE staining revealed that the villi of the jejunum were shortened and coarsened compared with those of the control, indicating an irregular striated border and desquamation of epithelial cells (Fig. 4C). Additionally, IHC analysis revealed clear brown positive signals of the PoRVA antigen on the villous epithelium of the intestine, whereas no such signals were observed in the control group piglets (Fig. 4D). These results indicate that the PoRVA strains QT2023 and BH2023 both successfully colonizes the intestinal cells of piglets, causing significant pathological damage.

## Discussion

Porcine rotavirus (PoRV) infection typically leads to diarrhea in piglets aged 1–5 weeks. However, in PoRV-positive pigs, diarrhea symptoms have been observed in neonatal piglets within 1–3 days of birth (data not shown). Given the prevalence of African swine fever virus, pig farms have enforced stringent biosecurity measures to reduce the horizontal transmission of diseases. Consequently, PoRV infection in newborn piglets is likely a result of vertical transmission from sows. In this study, the presence of PoRVA nucleic acid was identified in reproduction-related samples (Fig. 1B). PoRVA can inhabit the testicular tissue of neonatal suckling piglets and express viral proteins (Fig. 1C). The neonatal piglets were collected immediately after birth to prevent any potential oral transmission through the colostrum-oral or fecal-oral routes that could occur post-delivery. Furthermore, PoRVA strains can be isolated from the testicular tissue of neonatal piglets (Fig. 3). Oral administration of these isolates can induce typical diarrhea symptoms in suckling piglets (Fig. 4). These findings indicate that PoRVA can breach the placental barrier and infect newborn piglets through vertical transmission.

Conversely, porcine diarrhea viruses such as PEDV, PoRV, PDCoV, and TGEV primarily spread through the fecal‒oral route [22–24]. While TGEV and PDCoV are known to infect swine testicular (ST) cells, there have been no studies reporting their presence in reproductive organs. Notably, PEDV, another important porcine virus, has been confirmed in piglet testicles and umbilical cords from PEDV-positive sows [25]. This serves as direct evidence that PEDV is transmitted vertically through the placenta. The present study revealed that two genotypes of PoRVA have the potential for vertical transmission, suggesting that some well-known enteric viruses may unexpectedly exist in reproductive organs. This insight aids in understanding the transmission patterns of swine enteric viruses that cause diarrhea. While RV were detected in colostrum and umbilical cord blood, viruses shed into feces were still the major source of rotavirus infection. Boosting lactogenic immunity with vaccination is the most crucial strategy for delivering passive antibodies to neonates via colostrum and reducing virus shedding in sows [9, 26]. Additional approaches may include strict biosecurity measures along production (e.g., hygienic separation of age groups with hand washing, plastic boot covers, regular washing of fomites, controlled entry of stakeholders on premises, etc.) [27, 28].

The VP7 and VP4 RV antigens are known to induce neutralizing antibodies, which determine the G genotype and P genotype of RV, respectively [15]. In pigs, 12 G genotypes and 16 P genotypes of RVA have been identified [9]. G9 RVA strains have been identified in both humans and pigs, with pigs being considered a potential host reservoir for human G9 RVAs [29, 30]. The RVA G9P [23] genotype has been documented in various countries, including China, Korea, Japan, Thailand, Belgium, Italy, the U.S.A., and Brazil [31]. Recently, a G9P [23] strain was isolated from a child with severe diarrhea in Thailand, providing evidence of porcine-to-human interspecies transmission of the G9P [23] RVA strain [32]. In this study, we sequenced and characterized the complete genome of the PoRVA strain QT2023 (RVA/Pig/CHN/QT/2023/G9P [23]) obtained from diarrheic piglets on a pig farm in China. Comprehensive genome analyses revealed that this strain has a human-RVA-like genetic backbone and is classified into the G9-P [23]-I5-R1-C1-M1-A8-N1-T1-E1-H1 genotype (Table 2). It has been reported that the G9P [23] strain is the most prevalent PoRVA strain, particularly in regions with high pig populations [33]. Therefore, strain QT2023 may contribute to the development of effective preventive and therapeutic strategies against G9P [23] strain infections in piglets and prevent RVA transmission across species to humans.

RV G12 strains have been increasingly detected worldwide and are emerging as prominent human G genotypes [34]. The porcine strain RU172 (G12P [7]) was first identified in eastern India in 2002, marking the initial discovery of a porcine-origin G12 virus [35]. Through full genome analysis, this strain was classified as the G12-P [7]-I5-C1-M1-R1-A1-N1-T1-E1-H1 genotype. No other G12P [7] strain was reported in pigs until 2021, when a porcine G12P [7] strain (CN127) was identified in pig farms in China, exhibiting a genotype constellation of G12-P [7]-I1-C1-M1-R1-A8-N1-T1-E1-H1 with a Wa-like genetic makeup [15]. In this study, we present another porcine G12P [7] strain, BH2023 (RVA/Pig/CHN/BH/2023/G12P [7]), which has a unique genotype configuration: G12-P [7]-I5-R1-C1-M1-A8-N1-T1-E1-H1, distinct from the previously known G12P [7] strains. This discovery enhances our comprehension of the evolving rotavirus landscape and underscores the risk of the potential dissemination of the G12P [7] genotype of PoRVA among pigs.

Pig RVs frequently breach the species barrier into humans, leading to the emergence of many porcine-like human rotavirus strains through interspecies transmission involving genetic reassortment [14]. Therefore, pigs are recognized as potential host reservoirs for these emerging genotypes and serve as mixing vessels for RVs [15]. Strains QT2023 and BH2023 display a genomic backbone reminiscent of porcine origin (Table 2). The presence of the VP6 genotype I5 and NSP1 genotype A8 in strains QT2023 and BH2023 aligns with the common occurrence of these genotypes in porcine strains [8], reinforcing their classification within the porcine RV lineage. Intriguingly, phylogenetic analysis revealed a close relationship between multiple genes of QT2023 and BH2023 and the corresponding genes of human-origin viruses (Table 2; Fig. 2). Therefore, strains QT2023 and BH2023 may have emerged from genetic reassortment between porcine and human RVs, suggesting that the concurrent circulation of human and porcine strains could contribute to the genesis of new genotype RVs.

## Conclusions

This report reveals a high prevalence of PoRVA-positive rates in reproductive samples of pigs. The isolation of the G9P [23] and G12P [7] genotypes of PoRVA in neonatal piglet testicles highlights their infectivity in piglets. Our findings provide evidence that PoRVA can breach the placental barrier and spread to newborn piglets through vertical transmission. These discoveries offer valuable insights into the transmission route of porcine RVA and have the potential to guide the development of efficient vaccine strategies for combating this disease.

### Sequence determination and analysis

Specific amplification primers targeting the genes of PoRVA were designed on the basis of the conserved regions (Table 1). The primers were synthesized by Sangon Bioengineering Co., Ltd. in Shanghai, China. cDNA was generated via TransScript II First-Strand cDNA Synthesis SuperMix (TransGen, Beijing, China) using random primers. The full-length genes were amplified via PrimeSTAR Max DNA Polymerase (Takara, Dalian, China). All RT‒PCR products were subjected to gel electrophoresis on a 1.5% agarose gel. The target bands were cloned into the pEasy-Blunt cloning vector (TransGen, Beijing, China) and sequenced by Sangon Bioengineering Co., Ltd. Phylogenetic trees were constructed via the neighbor-joining method using MEGA7 software. The bootstrap values were calculated according to 1,000 replicates of the alignment.

### Evaluation of histology and immunocytochemistry

During necropsy, samples of testicular and intestinal tissues were collected and fixed with 4% paraformaldehyde for histopathological and immunohistochemical (IHC) examinations. For histopathological examination, tissue sections with a thickness of 3 μm were stained with hematoxylin and eosin (HE). The sections were first stained with hematoxylin for 5 min followed by eosin for 30 s. The sections were then observed and photographed under a microscope (Olympus BH-2).

To visualize the distribution of virus particles in the tissues, an IHC kit (Sangon, Shanghai, China) was used. Briefly, the paraffin sections were dewaxed with xylene and then gradually rehydrated in low-concentration ethanol. The sections were placed in 10 mM citrate buffer (pH 6.0) and heated in a pressure cooker for 30 min for antigen retrieval. An inactivating enzyme mixture was added, and the mixture was incubated at room temperature away from light for 15 min. Subsequently, 3% bovine serum albumin (BSA) in PBS with Tween (PBST) was added to block nonspecific binding sites at room temperature for 30 min. The sections were incubated overnight at 4 °C with an antibody against RVA VP6 protein (Rotavirus Omnitope polyclonal goat serum, ViroStat, Portland, ME) at a dilution of 1:100. The next day, the sections were brought to room temperature for 40 min and incubated with HRP-conjugated rabbit anti-goat IgG (Abbkine, A21030) at 37 °C for 30 min. Each section was stained with haematoxylin somatic cell fast staining solution for 5 min and rinsed with distilled water. The sections were then decolorized in a solution of 1% hydrochloric acid-ethanol for 3 s. After decolorization, the sections were rinsed in distilled water and placed in PBST to reverse blue for 5 min. Subsequently, the sections were subjected to a series of ethanol washes (75%, 85%, and 95%) for 5 min and then soaked in anhydrous ethanol for 5 min. The sections were soaked in xylene for 10 min. Then, neutral gum was added to the sections, and a glass slide was placed onto them. Throughout the IHC procedure, the sections were washed four times with fresh PBS-Tween. Finally, the sections were observed and photographed.

### Isolation of PoRVA strains

The fresh newborn piglet testicle was collected immediately after birth and sampled with 1 g pieces. Then the samples were carefully dissected into small pieces and transferred into a 15 mL tube with 5 mL serum-free Dulbecco’s Modified Eagle Medium (DMEM) (Thermo Fisher Scientific, Waltham, MA, USA), then centrifuged at 1,800 × *g* for 10 min at 4 °C. The supernatants were collected and filtered through the syringe filter with a 0.22 μm pore size. Then 200 µL supernatant was mixed with 200 µL DMEM including 5 µg/mL trypsin (Sigma-Aldrich, St. Louis, MO, USA) and inoculated onto the monolayers of MA-104 cells (American Type Culture Collection, ATCC), which were rinsed twice with PBS in advance. After incubation at 37 °C for 1 h with constant shaking, 1 mL maintenance media with 10 µg/mL trypsin were added and the plates were placed at 37 °C in an incubator with 5% CO_2_ for 48 h. Then the plates were treated with a freeze-thaw cycle twice to obtain the first passage (F1) viruses. After centrifuging at 1,800 × *g* for 1 min at 4 °C, the supernatant was inoculated onto the monolayers of MA-104 cells grown in T25 cell culture flask for further propagation until the cells developed a visible cytopathic effect (CPE).

### Pathogenicity experiments

Fifteen 7-day-old suckling piglets born to sows that tested negative for both PoRV antigen and antibody were randomly divided into three groups, each comprising 5 piglets. Two groups of piglets were housed separately in distinct pigpens and orally administered 1 × 10^6.0^ TCID_50_ of strains QT2023 (the 13th passage, P13) or BH2023 (P13), while the control group received an equivalent volume of saline. Fecal swabs were collected at 12, 24, 36, 48, and 60 hpi. After 60 hpi, the piglets were euthanized for necropsy. Samples from the small intestines were obtained to prepare pathological slices.

### Nucleotide sequence accession numbers

The nucleotide sequence of strain BH2023 was submitted to the GenBank database and assigned the accession numbers PP391045 (NSP1), PP391046 (NSP2), PP391047 (NSP3), PP391048 (NSP4), PP391049 (NSP5), PP391050 (VP1), PP391051 (VP2), PP391052 (VP3), PP391053 (VP4), PP391054 (VP6), and PP391055 (VP7). The accession numbers of QT2023 in the GenBank database are PP384134 (NSP1), PP384135 (NSP2), PP384136 (NSP3), PP384137 (NSP4), PP384138 (NSP5), PP384139 (VP1), PP384140 (VP2), PP384141 (VP3), PP384142 (VP4), PP384143 (VP6), and PP384144 (VP7).

## Data Availability

Sequence data that support the findings of this study have been deposited in the GenBank and the primary accession codes are provided within the manuscript.

## Abbreviations

PoRVA: Porcine group A rotavirus
RV: Rotavirus
DsRNA: Double-stranded RNA
VP: Viral proteins
NSP: Nonstructural proteins
qRT‒PCR: Reverse transcription qPCR
IHC: Immunohistochemical
BSA: Bovine serum albumin
PBST: PBS with Tween-20
TCID_50_: Median tissue culture infectious dose
ORF: Open reading frame
HE: Hematoxylin-eosin
PEDV: Porcine epidemic diarrhea virus
PDCoV: Porcine deltacoronavirus
TGEV: Transmissible Gastroenteritis Virus
